# Healthcare workers’ knowledge, attitude and practices on infection prevention and control in the context of the COVID-19 pandemic at the Faranah regional hospital and associated healthcare centers, Guinea

**DOI:** 10.1186/s13756-024-01435-z

**Published:** 2024-07-18

**Authors:** Lena Landsmann, Anna Borodova, Carlos Rocha, Aziz Amadou Diallo, Kamis Mamadou Diallo, Matthias Borchert, Mardjan Arvand, Mamadou Diallo, Rebekah R. Wood, Sophie A. Müller

**Affiliations:** 1 Unit for Hospital Hygiene, Infection Prevention and Control, Robert Koch Institute, Berlin, Germany; 2 Centre for International Health Protection, Robert Koch Institute, Berlin, Germany; 3 Faranah Regional Hospital, Faranah, Guinea

**Keywords:** COVID-19, SARS-CoV-2, Infection prevention and control (IPC), Healthcare workers (HCW), KAP, Hand hygiene (HH), COVID-19 response, Lower-Middle-Income Country (LMIC), Triaging, Screening, Alcohol-based handrub (ABHR), Guinea

## Abstract

**Background:**

In response to the COVID-19 pandemic, WHO launched a strategic preparedness and response plan, outlining public health measures to support countries worldwide. Healthcare workers have an increased risk of becoming infected and their behaviour regarding infection prevention and control (IPC) influences infection dynamics. IPC strategies are important across the globe, but even more in low-resource settings where capacities for testing and treatment are limited. Our study aimed to assess and implement COVID-19 pandemic preparedness and response measures in Faranah, Guinea, primarily focusing on healthcare workers’ IPC knowledge, attitude and practice (KAP).

**Methods:**

The study was conducted between April 2020 and April 2021 assessing IPC pandemic preparedness and response measures such as healthcare workers’ KAP, alcohol-based handrub (ABHR) consumption and COVID-19 triaging in the Faranah Regional Hospital and two associated healthcare centres. The assessment was accompanied by IPC training and visual workplace reminders and done in pre- and post- phases to evaluate possible impact of these IPC activities.

**Results:**

The overall knowledge score in the Faranah Regional Hospital was 32.0 out of 44 at baseline, and did not change in the first, but increased significantly by 3.0 points in the second follow-up. The healthcare workers felt closer proximity to SARS-CoV-2 overtime in addition to higher stress levels in all study sites. There was significant improvement across the observed triaging practices. Hand hygiene compliance showed a significant increase across study sites leading to 80% in Faranah Regional Hospital and 63% in healthcare centers. The average consumption of ABHR per consultation was 3.29 mL with a peak in February 2020 of 23 mL.

**Conclusion:**

Despite increased stress levels among HCWs, the ongoing IPC partnership well prepared the FRH in terms of triaging processes with a stronger impact on IPC practice than on theoretical knowledge. Throughout the pandemic, global shortages and surges in consumption did not affect the continuous ABHR provision of the FRH. This highlights local ABHR production as a key pandemic preparedness strategy.

**Supplementary Information:**

The online version contains supplementary material available at 10.1186/s13756-024-01435-z.

## Background

In December 2019, the People’s Republic of China reported a cluster of pneumonia that was later identified as Coronavirus Disease 2019 (COVID-19), which is the clinical manifestation of severe acute respiratory syndrome Coronavirus 2 (SARS-CoV-2) infection. The World Health Organisation (WHO) declared the COVID-19 outbreak a pandemic on March 11, 2020 [1] and launched a strategic preparedness and response plan, outlining public health measures to support countries worldwide, and highlighting the importance of scaling up infection prevention and control (IPC) capacities [2]. Healthcare workers (HCWs) are responsible for the management of COVID-19 patients, and therefore have an increased risk of becoming infected and their IPC behavior can influence infection dynamics in the healthcare setting while at work, and in the community when home. The occupational risk to contract the disease varies between healthcare settings, with healthcare associated outbreaks among HCWs being previously described [3]. While the implementation of an appropriate IPC strategy to contain the spread of SARS-CoV-2 is of great importance everywhere, this is particularly evident in low-resource healthcare systems, where capacities for testing and treatment of COVID-19 are limited [4–6].

In Guinea, the first case of COVID-19 was documented on March 5, 2020 [7] and then followed by containment measures, such as a 14 day monitoring of travellers from high-risk areas, the closure of Conakry airport, and a ban on gatherings of more than 100 people in the capital Conakry [8, 9]. On March 30, more measures were implemented, such as a curfew and a ban on movements from Conakry to the interior of the country [8, 9]. Up to December 22, 2023, Guinea has recorded 38,572 confirmed cases and 468 deaths [10].

Reliable access to personal protective equipment (PPE), adherence to IPC measures and early detection of SARS-CoV-2 infection have been shown to be determining factors in containing the spread of the disease and reducing HCWs’ risk of infection [11, 12]. Our study aimed to assess and implement COVID-19 pandemic preparedness and response measures in Faranah, Guinea. Our measures primarily focused on HCWs’ IPC knowledge, attitude and practice (KAP), in order to tailor IPC capacity improvement strategies through needs-based training and implementation of context specific evaluation tools.

## Methods

### Study site

The study was conducted in the framework of the PASQUALE project (Partnership to Improve Patient Safety and Quality of Care), a long-term partnership between the Faranah Regional Hospital (FRH) (*Hôpital Régional de Faranah*) and the Robert Koch Institute (RKI) in Berlin. The study was carried out in the FRH and two healthcare centres (HCCs), Abattoir (urban) and Tiro (rural). The overall project goals are to increase patient safety through improvement of hand hygiene (HH), including the implementation of local production of alcohol-based handrub (ABHR), and the introduction of the Surgical Safety Checklist (SSC) based on the WHO Global Patient Safety Challenges “Clean care is safer care” and “Safe surgery saves lives” [13, 14].

The FRH is a reference hospital for a population of over 300,000 inhabitants, employing around 100 healthcare and administrative staff members, and providing essential care with a capacity of 80 beds, one operating theatre for general and trauma surgery, one operating theatre for gynaecology and obstetrics and, in the aftermath of the West African Ebola epidemic 2013–2016, an isolation ward. At the beginning of the pandemic, the FRH established a COVID-19 triage zone at the entrance of the outpatient and emergency department, near the main entrance of the hospital. The triaging process was defined as screening for COVID-19 suspect symptoms (such as fever and respiratory symptoms) according to the guidelines of the national COVID-19 task force set up by the National Agency for Health Security (*Agence Nationale de la Sécurité Sanitaire*, ANSS) [15].

HCC Tiro is located in a rural area at 40 km distance from the FRH and employs 11 HCWs, while HCC Abattoir is located in the urban setting of Faranah prefecture, employing 49 HCWs. HCCs provide healthcare services for common health problems such as testing and treatment of malaria, prenatal care, spontaneous delivery, and vaccination, but no surgical procedures [16]. Both centres are supplied with ABHR from the FRH.

### Study population

All HCWs employed by the FRH and the two HCCs at the time of the study were eligible and invited to participate. Informed written consent was obtained.

### Study design

**Fig. 1:**
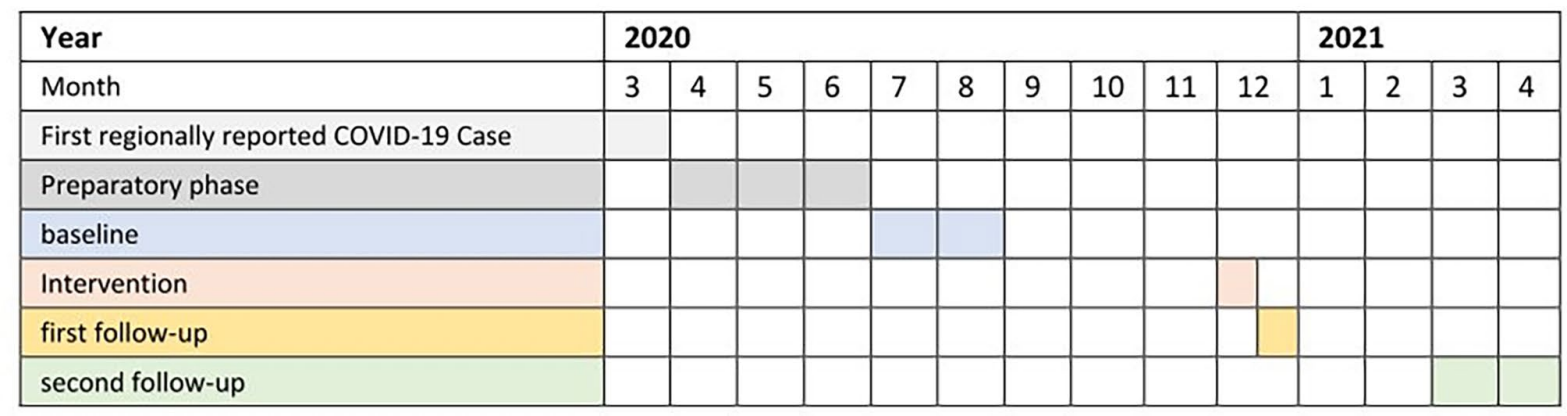
Study Timeline

The study was conducted from April 2020 to April 2021 (Fig. 1). Our study aimed to enhance our understanding of the pandemic preparedness and response capacities of the FRH in relation to the ongoing PASQUALE IPC project. The study specifically assessed HCWs’ KAP on IPC, continued local production of ABHR and implementation of a COVID-19 triage process. A needs-based IPC training and workplace reminders were also conducted to facilitate improvements in IPC KAP and culture. In addition, we carried out a qualitative study for a more in-depth assessment of HCW’s beliefs and attitudes (Data under submission).

Our study consisted of five phases, including three assessment periods: (1) a preparatory phase, (2) a baseline KAP assessment of IPC and COVID-19 triage, (3) a training on IPC and COVID-19 triage, (4) a first immediate post-training follow-up assessment of IPC knowledge and attitude only, and (5) a second follow-up assessment three months post-training of all IPC KAP components and COVID-19 triage. HH observations were not done immediately after the training so as to give room for indirect knowledge and practice transfer as all HCWs were viable for observation independent of their training participation. The knowledge and attitude assessment were carried out using a questionnaire adapted from a previous study [17]. The implementation of the triage algorithm included the use of PPE and the screening for suspected COVID-19 cases, and was assessed by direct observations using an observation form designed by the study team based on recommendations by the *Centers for Disease Control and Prevention* (CDC) [18]. HH compliance was assessed using the WHO observation form for “5 Moments of Hand hygiene” [19] that has been used at the hospital repeatedly since the beginning of the PASQUALE project in 2017 [20–22], with two additional HH indications for this study: “before use of PPE” and “after use of PPE”. These two indications were covered in the training.

The intervention consisted of an in-person training provided by a national trainer, who previously had received training on COVID-19 by the ANSS, comprising theoretical and practical sessions on IPC and COVID-19 triage. We placed posters as reminders in the workplace for PPE and HH.

ABHR consumption was defined as the quantity of ABHR in mL requested by staff at the hospital’s pharmacy, and was assessed on a monthly basis. In order to assess ABHR consumption per consultation, the monthly consumed ABHR in mL was divided by number of consultations in respective months.

### Data collection and statistical analysis

All data was entered in Epi Info (Version 2.2.3.0) and analysed using STATA Standard Edition (StataCorp LCC Version 17). Since FRH and the HCCs are at different levels in the Guinean health care system with different HCW composition, their data were analysed separately, while data of the two HCCs, Abattoir and Tiro, were combined. In HCCs and the FRH nursing and medical students as well as technicians were combined into the “other” category.

We conducted a paired analysis of KAP questionnaire results to assess the impact and significance of training by comparing those who participated in all assessment periods. A knowledge score was calculated where each “correct” answer was worth 1 point (maximum score: 44 points). Since knowledge scores across all periods were not normally distributed, they were described as medians and interquartile ranges (IQR), and compared using the Wilcoxon matched-pairs signed-rank test. Likert scale (1 = completely disagree to 7 = completely agree) attitude responses across periods were analyzed using the Wilcoxon matched-pairs signed-rank test with median and IQR reporting. Six attitude questions (32, 34, 35 and 38a-c) were chosen to be described in detail according to relevance. Two-tailed p-values less than 0.05 were considered to be statistically significant throughout all analyses and statistical tests.

Observations on IPC practices concerning triage for symptoms and correct PPE usage among HCWs were conducted at baseline and in the second follow-up assessment. Based on CDC guidelines [23], the observer checked “yes” if actions related to proper IPC indications were performed: (1) patient’s temperature checked, (2) distance from patient maintained, (3) HCW wearing PPE, (4) patient evaluated for respiratory conditions. Baseline was compared to follow-up with χ^2^ tests and a design effect of two was used to account for a lack of independence in observations [24]. The same analysis was done for PPE usage, i.e. (1) type of mask and (2) correct usage of mask [25]. We excluded “patients presenting respiratory symptoms wear masks” from the analysis, since the observer could not assess the presence of respiratory symptoms by direct observation only, thus no statement could be made regarding the correct indication and application. As wearing of goggles, gloves and blouse was recommended for “patients presenting respiratory symptoms” only, these types of PPE were not included in the analysis and “HCW wearing PPE” reflects mask use only.

HH compliance was assessed via direct overt observations by a local research assistant of PASQUALE, known to the HCWs. The compliance rate following the “5 Moments of Hand Hygiene” in % was calculated as the number of HH actions performed, divided by the number of all opportunities requiring HH action [26] as previously described by the study team [21]. For better comparison with previous HH studies of the project and with other international studies, the two additional indications of PPE usage were analyzed separately and not included in the overall compliance (Supplementary Table 2). Baseline was compared to each follow-up by χ^2^ tests and a design effect of two was used to account for lack of independence. Multivariable logistic regression was performed with HH compliance as the outcome and period number as the independent variable. Confounders that were found in previous study phases [21], such as “hand hygiene indication” and “professional category” were included in the final logistic regression model if the crude OR differed substantially from the adjusted one.

## Results

### Study demographics

In FRH and HCCs, 32 HCWs and 16 HCWs took part in all three assessment periods, respectively (Supplementary Table 1), whereby the majority of HCWs were auxiliary nurses, followed by nurses and others, such as medical and nursing students as well as technicians. The majority of HCWs had 6 + years of experience (FRH: 71.9%, HCCs: 56.3%). While 21.8% in FRH had received previous COVID-19 IPC training at baseline, none of the HCWs in HCCs. FRH HCWs reported in the baseline assessment that their primary sources of COVID-19 information were ‘word of mouth’ (81.3%), the FRH itself (81.3%), ‘television, radio or magazines’ (75.0%) and ‘social media’ (75.0%). HCC HCWs reported ‘television, radio or magazines’ (93.8%), ‘social media’ (87.5%) and ‘word of mouth’ (68.8%) as primary information sources in baseline.

### COVID-19 IPC knowledge scores

#### Faranah Regional Hospital

The overall knowledge score in FRH was 32.0 at baseline (Table 1) and did not change in the first follow-up (± 0) but increased significantly by 3.0 points in the second follow-up (*p* = 0.029).

No professional groups showed improvement upon first follow-up, but medical doctors, midwives and “others” showed considerable decrease in knowledge. The only significant improvement was seen for auxiliary nurses at second follow-up.

#### Healthcare centres

In HCCs, the knowledge score increased considerably upon first follow-up and remained at this level in second follow-up; however, this improvement over baseline failed to be statistically significant by a small margin only, with a p-value of 0.051 (Table 1).

**Table 1:** Median IPC knowledge score (IQR); maximum score: 44

Faranah Regional Hospital							
		Baseline	Follow-up 1 Difference to baseline	p*	Follow-up 2 Difference to baseline	p*	p#
Overall Knowledge Score		32.0 (30.0–36.0)	+ 0.0	0.017	+ 3.0	0.029	< 0.001
By professional categories							
	Medical doctor	37.5 (36.5–39.5)	− 3.5	0.250	+ 1.0	0.875	0.125
	Auxiliary Nurse	31.0 (28.0–32.0)	+ 0.0	1.0	+ 5.0	0.003	0.004
	Nurse	32.0 (28.0–36.0)	+ 0.0	0.781	+ 1.0	0.219	0.438
	Midwife	33.0 (26.0–25.0)	− 5.0	0.750	+ 0.0	1.0	0.500
	Other	36.0 (30.0–40.0)	− 3.0	0.016	+ 0.0	0.98	0.047

**Healthcare Centers**							
		Baseline	Follow-up 1 Difference to baseline	p*	Follow-up 2 Difference to baseline	p*	p^#^
Overall Knowledge Score		31.0 (29.0-33.5)	+ 3.5	0.099	+ 4.0	0.051	0.419

* p-value calculated with Wilcoxon signed-rank test compared to baseline; # compared to follow-up 1

### Attitude

#### Faranah Regional Hospital

At baseline, the majority of HCWs fully agreed to the statement to “I know how to protect myself from getting infected” without significant change at both follow-ups (*p* = 0.242; *p* = 0.249 respectively) (Table 2). While at baseline only few HCWs felt that “SARS-CoV-2 is close to me”, there was a steady increase during first and second follow-up. The majority of HCWs agreed that “SARS-CoV-2 makes me feel helpless” at baseline, this decreased significantly at second follow-up when compared to baseline (*p* = 0.005). At baseline the majority of HCWs disagreed with the statement “SARS-CoV-2 makes me feel stressed”, however the feeling of stress increased significantly at second follow-up when compared to baseline (*p* < 0.001).

**Table 2:** Median attitude (IQR), Faranah Regional Hospital; likert scale 1–7 (1 fully disagree, 7 fully agree)

	Baseline	First follow-up Difference to baseline	*p* *	Second follow-up Difference to baseline	*p* *	*p* ^#^
I think I will become seriously ill, if I am infected with SARS-CoV-2	7.0 (3.5-7.0)	± 0	0.994	-1.0	0.181	0.336
I know how to protect myself from SARS-CoV-2	7.0 (7.0–7.0)	± 0	0.242	± 0	0.249	0.070
SARS-CoV-2 is close to me	2.0 (1.0–7.0)	+ 3.5	0.136	+ 4.0	0.020	0.326
SARS-CoV-2 makes me feel helpless	7.0 (5.5-7.0)	± 0	0.402	-2.5	0.005	0.010
SARS-CoV-2 makes me feel stressed	2.0 (1.0-6.5)	± 0	0.378	+ 4.0	< 0.001	0.002

* p-value calculated with Wilcoxon signed-rank test compared to baseline; # compared to first follow-up

#### Health Care centers

In the HCCs, the majority of HCWs agreed at baseline to the statement “I know how to protect myself”, which increased significantly after the intervention at first follow-up (*p* = 0.004). While only few HCWs at baseline felt that “SARS-CoV-2 is close to me”, this changed to the majority of HCWs in both follow-ups (Table 3). Compared to baseline, there was a significant increase of HCWs responding “SARS-CoV-2 makes me feel stressed” at second follow-up (*p* = 0.001).

**Table 3:** Median attitude (IQR), Health Care Centers; Likert scale 1–7 (1 fully disagree, 7 fully agree)

	Baseline	First follow-up Difference to baseline	*p* *	Second follow-up Difference to baseline	*p* *	*p* ^#^
I think I will become seriously ill, if I am infected with SARS-CoV-2	5.0 (1.5-7.0)	+ 2.0	0.016	+ 1.0	0.563	0.076
I know how to protect myself from SARS-CoV-2	6.0 (2.5-7)	+ 1.0	0.004	+ 0.5	0.270	0.005
SARS-CoV-2 is close to me	2.0 (1.0–7.0)	+ 5.0	0.007	+ 4.0	0.108	0.697
SARS-CoV-2 makes me feel helpless	6.0 (2.0–7.0)	+ 1.0	0.008	± 0	0.852	< 0.001
SARS-CoV-2 makes me feel stressed	1.0 (1.0–3.0)	+ 1.5	0.057	+ 5.0	0.001	0.142

* p-value calculated with Wilcoxon signed-rank test compared to baseline; # compared to first follow-up

### Triage observation at Faranah Regional Hospital and Healthcare Centers

The triage zone consisted of a desk at the entrance of the outpatient department with a HH station and a team of HCWs to perform a temperature and respiratory symptom check. During baseline and follow-up in FRH and HCCs a total of 713 observations of the triage processes were conducted (Table 4). All four categories of triaging practices (Temperature checked, Distance maintained, HCW wearing PPE, Respiratory condition evaluated) had significant improvements when comparing second follow-up to baseline in FRH and HCCs. The highest improvement was seen for PPE use increasing from 2.7 to 99.2% in FRH (*p* < 0.001) and 2.4–100.0% in HCCs (*p* < 0.001). FFP2/N95 masks were never used at any of the health facilities, whereas at HCCs there was a change in use of cotton masks to surgical masks. In second follow-up no cotton mask was used, but surgical mask usage increased to 98.8%. At second follow-up all HCWs used the masks correctly (100%) across all study sites.

**Table 4:** Triage process in Faranah Regional Hospital and Healthcare Centers

		FRH			HCC		
		Baseline N (%)	Follow-up N (%)	p*	Baseline N (%)	Follow-Up N (%)	p*
Total Observations		293	255		85	80	
Temperature checked		185 (63.1)	242 (94.9)	< 0.001	73 (85.9)	80 (100.0)	< 0.014
Distance maintained		129 (44.0)	254 (99.6)	< 0.001	0	70 (87.5)	< 0.001
HCW wearing masks		8 (2.7)	253 (99.2)	< 0.001	2 (2.4)	80 (100.0)	< 0.001
Respiratory condition evaluated		90 (30.7)	175 (68.6)	< 0.001	8 (9.4)	38 (47.5)	0.001
Mask usage							
	Surgical	293 (100.0)	252 (98.8)	0.188	11 (13.4)	79 (98.8)	< 0.001
	FFP2/N95	0	0		0	0	
	Cotton/Other	0	0		63 (76.8)	0	
	None	0	3 (1.2)		8 (9.8)	1 (1.2)	
	Correct usage of Mask	283 (96.6)	255 (100.0)	0.035	66 (79.5)	80 (100.0)	0.002

* p-value calculated with Wilcoxon signed-rank test compared to baseline

### Hand Hygiene Compliance

#### Faranah Regional Hospital

**Fig. 2:**
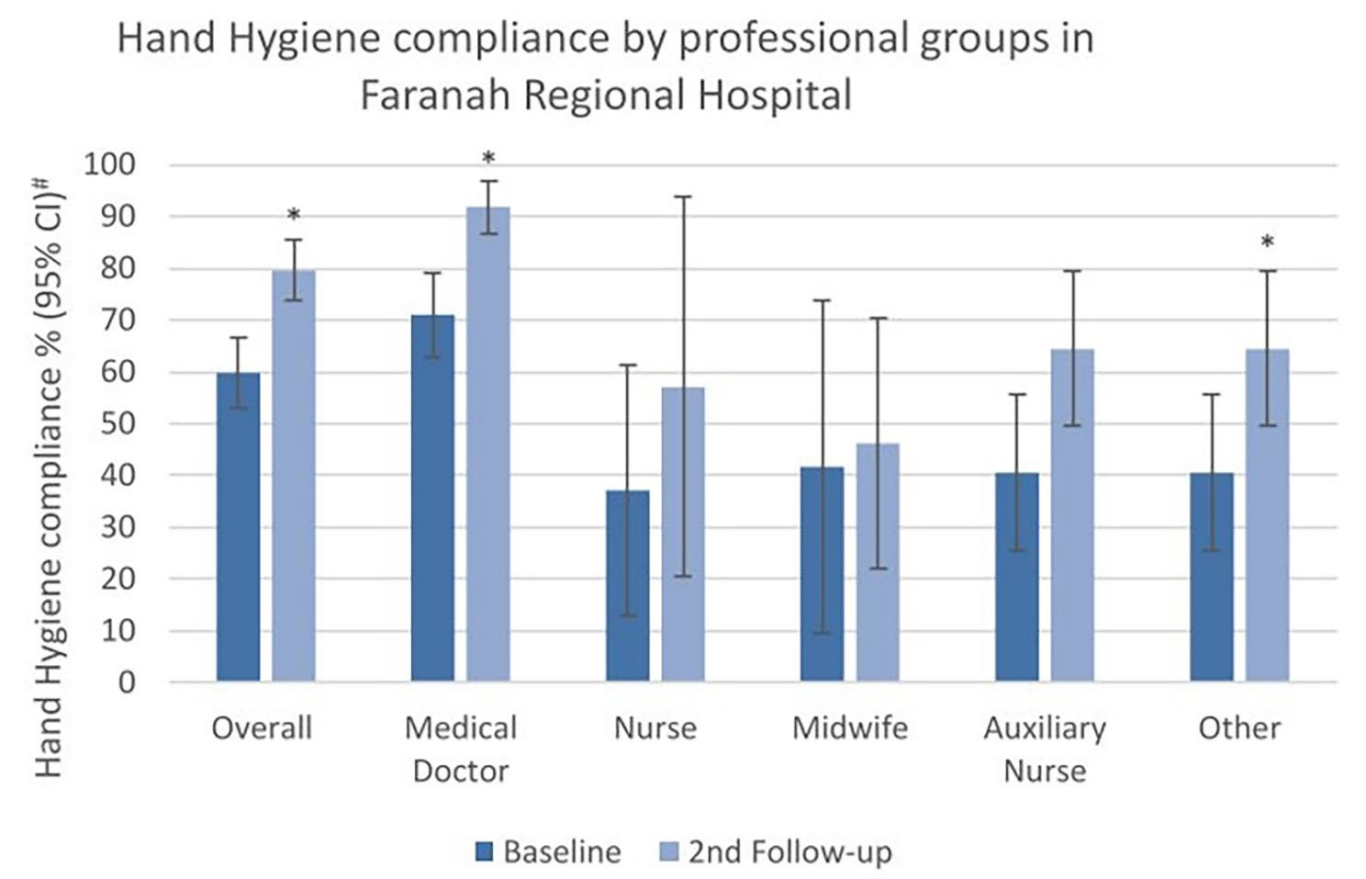
Hand Hygiene compliance (%) at baseline and second follow-up, overall and by professional categories in the Faranah Regional Hospital. # Width of CI adjusted for lack of independence by inflating standard error by a factor of 2. *p-value < 0.05 determined by x² test with standard error corrected by factor 2 to adjust for lack of independence

A total of 1,520 HH opportunities were observed in FRH with 774 at baseline and 746 at second follow-up. The overall compliance increased significantly from 59.8% at baseline to 79.6% at second follow-up (*p* < 0.001). There was an increase in compliance by approximately 20% points among all professional groups, except for midwives who showed the lowest increase of around 5% points. The most prominent increase was seen in medical doctors with the highest baseline and follow-up compliance (Fig. 2).

**Fig. 3:**
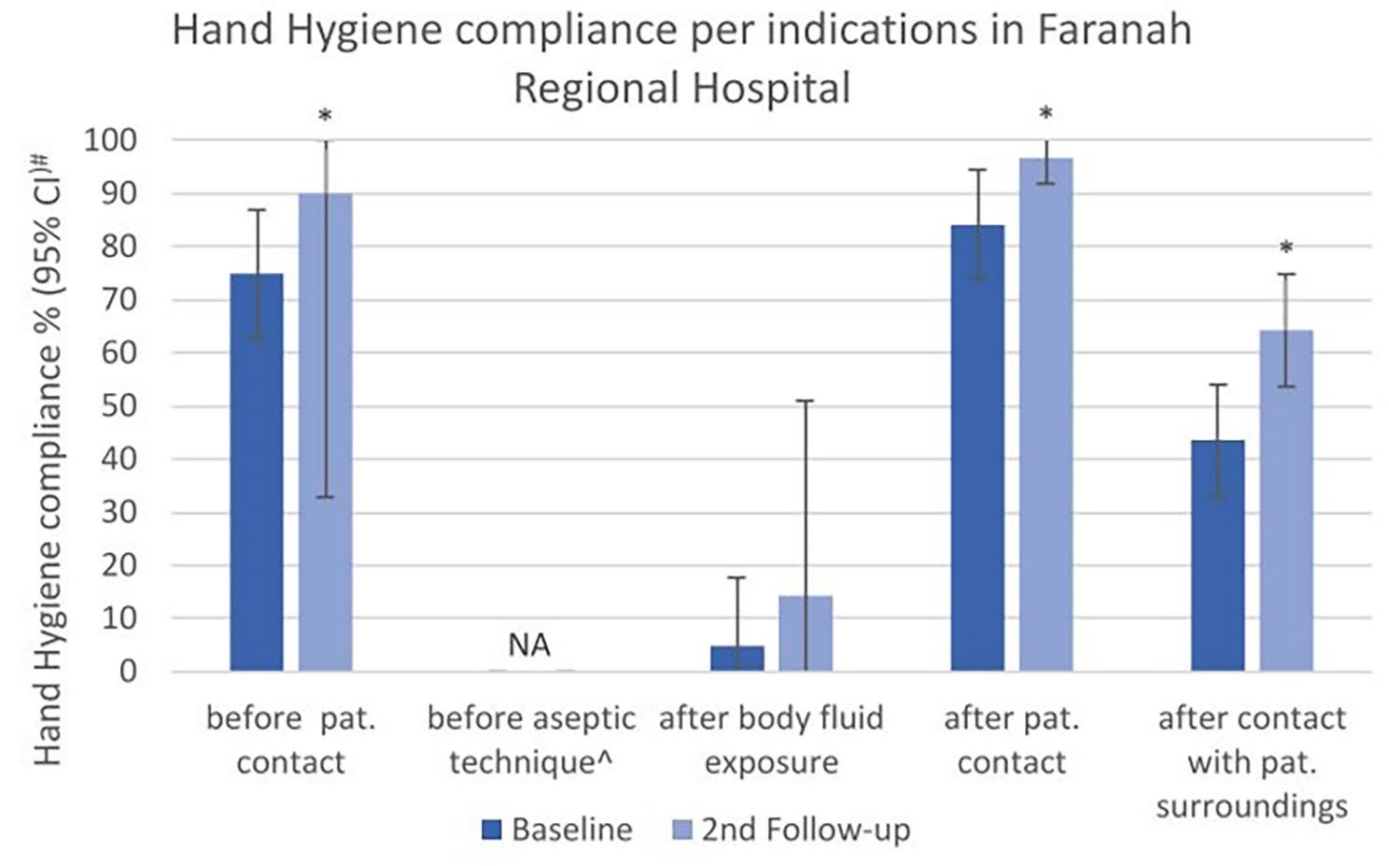
Hand Hygiene compliance (%) at Baseline and 2nd Follow-up, overall and by indication categories in the Faranah Regional Hospital. #Width of CI adjusted for lack of independence by inflating standard error by a factor of 2. * p-value < 0.05 determined by x² test with standard error corrected by factor 2 to adjust for lack of independence. ^For the indication “before aseptic technique” no opportunity was observed

Among indications, there was no opportunity observed for the indication “before aseptic technique”. The indication “after contact with patient surroundings” was the most observed, with a significant compliance increase from 43.6 to 64.3% (*p* < 0.001). The lowest compliance was observed for “after body fluid exposure” with 4.8% at baseline with a non-significant increase to 14.3% (*p* = 0.397) (Fig. 3). Complementary indications “before use of PPE” and “after use of PPE” did not improve significantly (Supplementary Table 2).

The multivariable analysis showed that increase in compliance was associated with the intervention (crude OR 2.62; 95% CI 1.66–4.15; *p* < 0.001). This association became stronger and remained significant after adjusting for the confounder of profession and for indication (adjusted OR 3.15; 95% CI 1.90–5.20; *p* < 0.001).

#### Healthcare centres

**Fig. 4:**
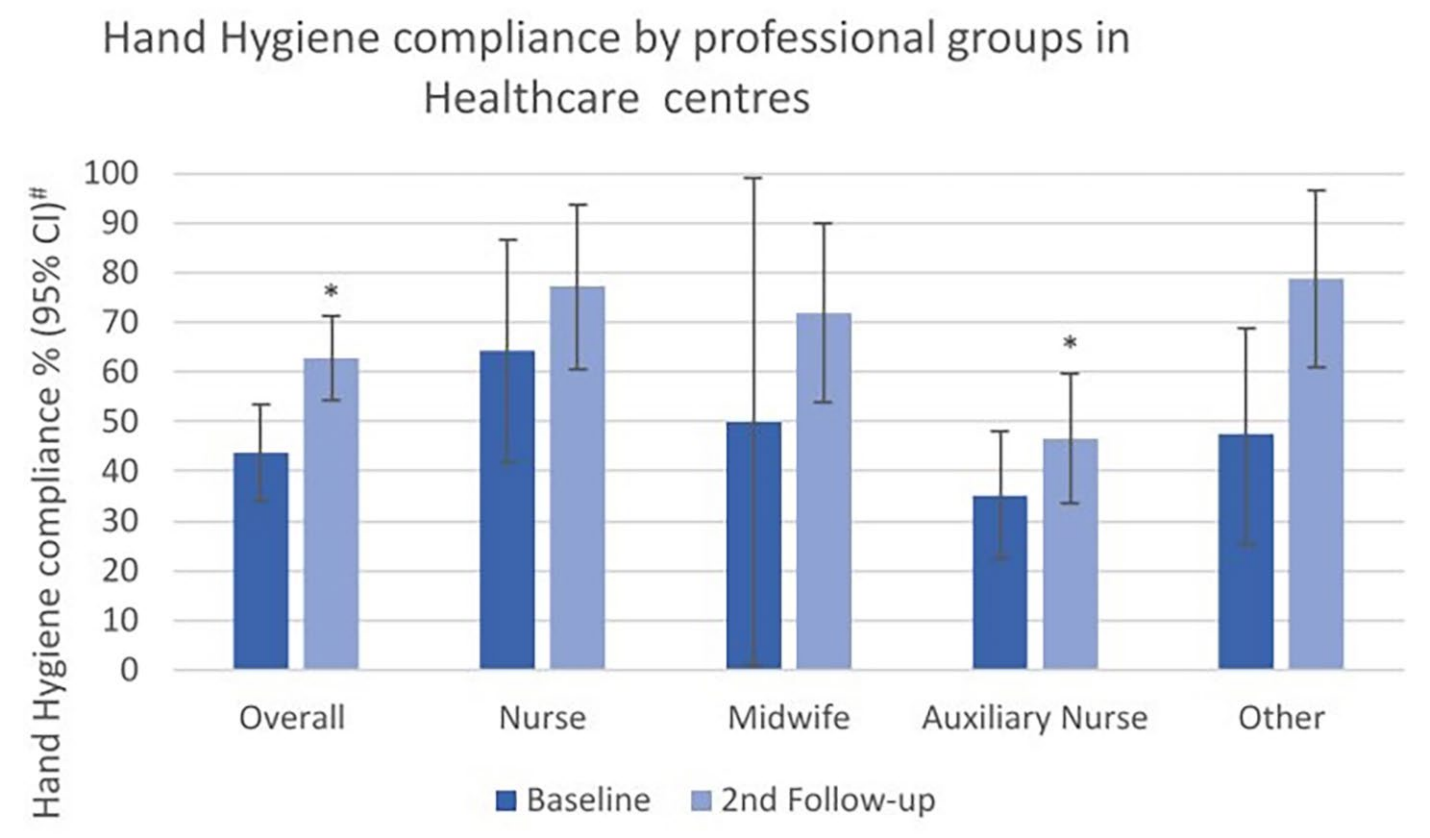
Hand Hygiene compliance (%) at baseline and second follow-up, overall and by professional categories in healthcare centers. #Width of CI adjusted for lack of independence by inflating standard error by a factor of 2. *p-value < 0.05 determined by x² test with standard error corrected by factor 2 to adjust for lack of independence

880 HH opportunities were observed in Abattoir and Tiro, with 386 at baseline and 494 at second follow-up. Overall compliance increased from 43.8 to 62.8% (*p* < 0.001). Compliance improved considerably among all professional groups and was significantly higher in “others” (*p* = 0.004, Fig. 4).

**Fig. 5:**
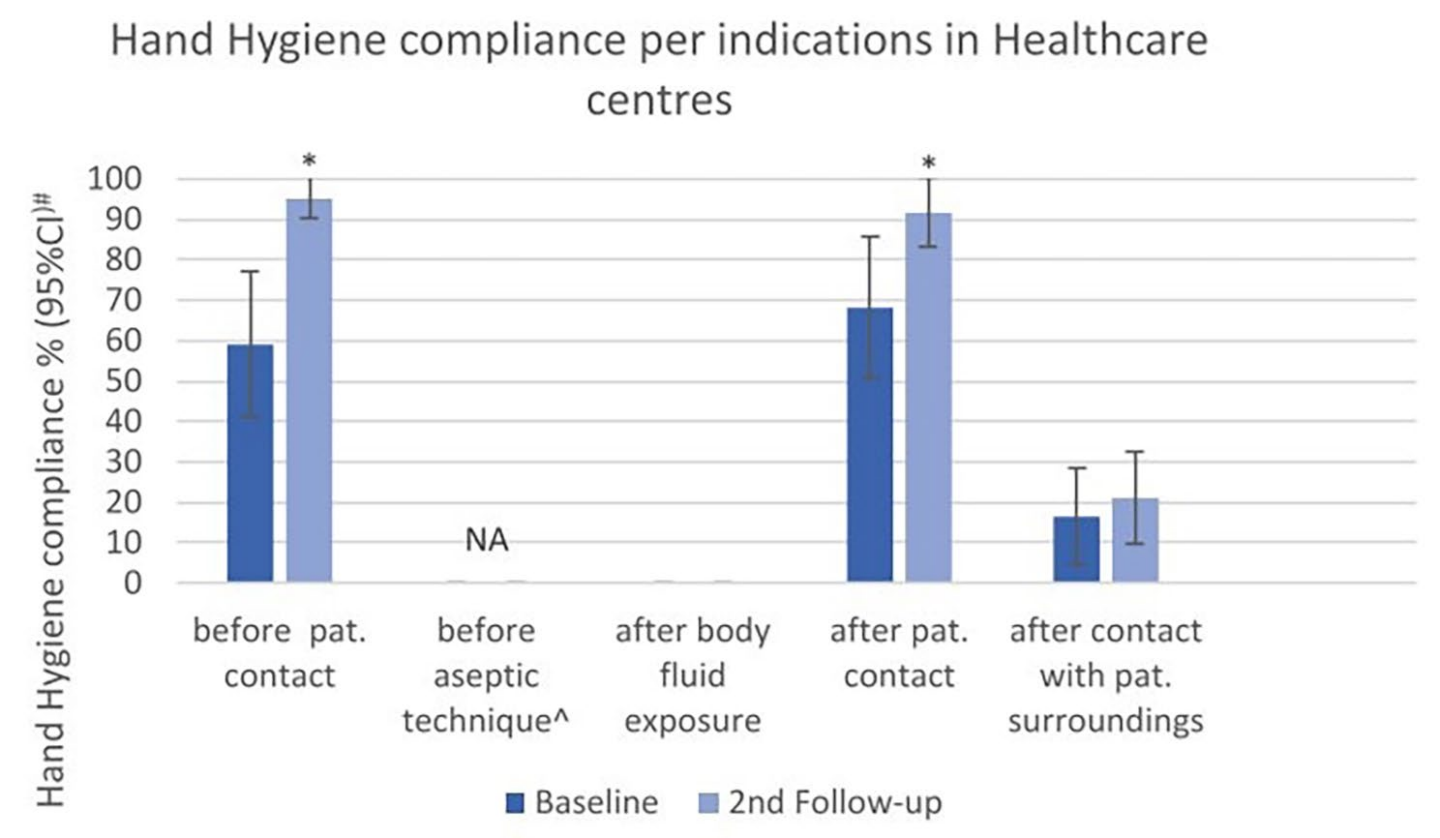
Hand Hygiene compliance (%) at baseline and second follow-up overall and by indications in healthcare centers. #Width of CI adjusted for lack of independence by inflating standard error by a factor of 2. *p-value < 0.05 determined by x² test with standard error corrected by factor 2 to adjust for lack of independence. ^The indication “before aseptic technique” was not applicable, as there was no opportunity observed

The highest significant improvement was observed for “before patient contact” with a compliance increase from 59.0 to 95.2% (*p* < 0.001). The lowest compliance was observed for “after body fluid exposure” with a baseline compliance of 0% and no change in second follow-up. There was no opportunity observed for the indication “before aseptic technique” (Fig. 5). The multivariable analysis showed that increase in compliance was associated with the intervention (crude OR 2.16; 95% CI 1.26–3.73; *p* < 0.001). This association became stronger and remained significant after adjusting for the confounder of each indication (adjusted OR 3.87; 95% CI 1.81–8.25; *p* < 0.001).

### ABHR Consumption

#### Alcohol-based handrub

**Fig. 6:**
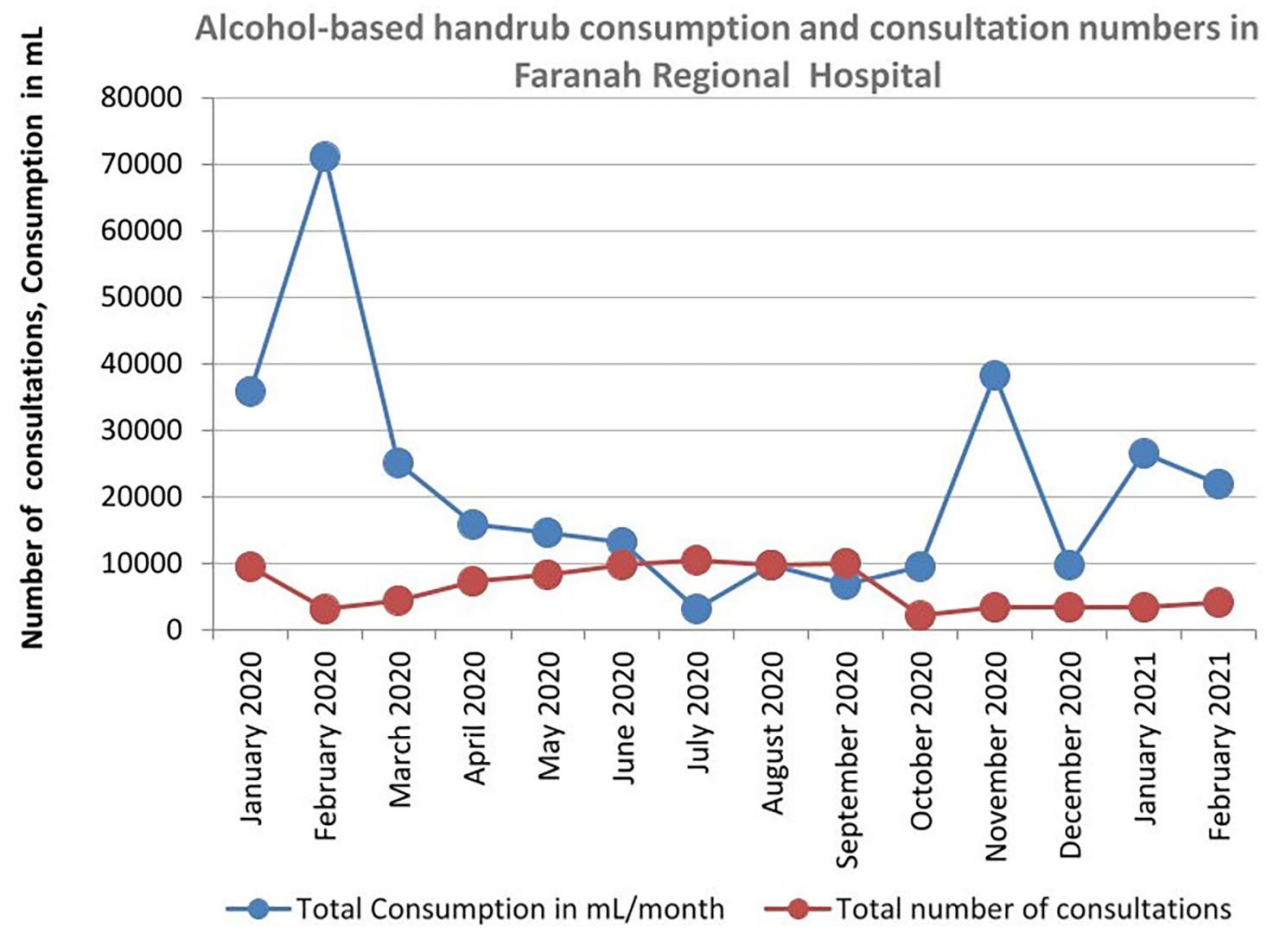
Monthly alcohol-based handrub consumption in mL/patient consultation in Faranah Regional Hospital

The monthly average ABHR consumption was 21.6 L with a monthly consultation average of 6,567, equaling to 3.29 mL of ABHR usage per consultation. The highest ABHR consumption was seen in February 2020 marking the beginning of the pandemic in Guinea with 23 mL ABHR used per consultation (Fig. 6).

## Discussion

This study was conducted to assess pandemic preparedness and IPC response aspects of the FRH in relation to the ongoing PASQUALE IPC project and COVID-19 pandemic with the aim of strengthening IPC capacities through implementation of a COVID-19 triage algorithm, needs-based training, and workplace reminders. HCWs’ KAP was assessed to measure the effectiveness of the training.

In the FRH the overall knowledge did not increase in first follow-up but showed significant improvement in second follow-up when compared to baseline. In HCCs the overall knowledge score increased in all assessment periods. High baseline knowledge among HCWs in the FRH could be due to regular hospital staff updates on COVID-19 and previously received COVID-19 training. The lack of improvement in first follow-up contrasts with previously published studies in FRH in which HCWs knowledge gain was greatest immediately after the training but showed signs of waning in the long-term [21]. However, literature from Guinea underlined that training does not necessarily lead to knowledge improvement [27]. Unchanged and decreased knowledge among HCWs in the FRH at first follow-up may be due to the training time period during which a wide range of information from various sources, including misinformation, was circulated. The anthropological observation in 2021 reported denial of the presence and actual emergence of a new and deadly virus in Guinea, as well as the belief that COVID-19 was a punishment from god (data under submission). Increased knowledge at second follow-up compared to baseline could reflect improved global COVID-19 knowledge and evidence that circulated in the health sector [28]. A study on COVID-19 misinformation circulation showed that ‘questionable’ and ‘conspiracy’ information peaked on Twitter around December 2020 [29], at the same time as our first follow-up. Therefore, it could stand to reason that this circulating misinformation was influential to affect knowledge. This rational was supported also by FRH and HCC HCWs reporting social media as one of their main information sources. Interestingly, on average the knowledge at baseline was not much lower at HCC than at FRH. But, in HCCs, the training was followed by an increase of knowledge across both follow-ups, suggesting that sustainable knowledge improvement happened over time. This increase likely occurred more strongly for HCC HCWs because they reported a complete lack of previous COVID-19 trainings.

Attitudes in both FRH and HCCs showed that the training increased the awareness of SARS-CoV-2 proximity and that SARS-CoV-2 induced stress increased over time. Higher stress levels might be linked to factors such as increased HCWs’ workload, pandemic awareness and fatigue [30], as well as lack of access to vaccines and vaccination hesitancy [31], as vaccination rates in the African continent were the lowest worldwide [32, 33]. External factors such as worsening political instability throughout the study could have also been a cause of increased stress levels [34]. Similar findings about the psychological burden of the COVID-19 pandemic on HCWs and the general population have already been reported in other studies [30, 35, 36]. Moreover, a study from Italy showed that HCWs who lacked access to and training on PPE use had a higher risk perception and thus were more likely to feel uncomfortable in handling patients with COVID-19 symptoms [37]. In our study, HCWs felt confident in protecting themselves, but feeling of stress increased over time. We conclude that the lack of access to recommended PPE, such as masks in the Faranah Region could have contributed to these reported feelings of stress in our study.

Triage performance improved in the FRH and associated HCCs. At baseline the use of masks had a very low compliance of 2.7% potentially reflecting the lack of availability as only very small quantities were in stock at the FRH. The following increase in mask use can be due to national recommendations, which made hospital mask usage mandatory. The second largest practice improvement in “maintaining the distance” can be explained by the training, as well as by global social distancing campaigns [38].

Significant increase in HH compliance emphasizes the positive effect of continuous trainings, a fact already demonstrated during the Ebola epidemic 2014–2016 [39]. This improvement is also enhanced with perceived increased risk and disease burden [40], which may have contributed to the improvement of IPC practices at second follow-up in our study, when the incidence in Guinea was at its maximum [41].

In terms of motivators for HH, a previous study showed differences in HCWs’ motivation in terms of patient protection versus self-protection [42], whereby in our study, HH both for the self-protection indication “after patient contact” and for the patient protection indication “before patient contact” significantly improved. We can thus conclude that patient safety and HCW safety were both important motivations to HCWs across study sites. Lack of compliance with the HH indication “after body fluid exposure“ can likely be explained by the fact that this is an indication requiring hand washing instead of hand rubbing when hands are visibly soiled [19]. Hand washing is challenging as neither the FRH nor the HCCs have ubiquitous sinks with running water. Therefore, HCWs need to leave the ward or the consultation rooms to perform hand washing at an improvised hand washing station with a canister. This finding emphasizes the need for critical facility IPC improvements in the Faranah region. In addition, the use of gloves and “double gloving” was observed for the HH indication “after body fluid exposure“, but HH actions after removing or changing gloves were not performed. The indication “after contact with patient surroundings”, has traditional low compliance and understanding [21], but over time made considerable gains. This indication differs from the others and has shown itself to be complex to understand what constitutes a “patient’s surroundings”, as this is the only indication without direct patient contact [19]. However, this indication accounts for the most opportunities with the second lowest compliance, emphasizing the need for training on different types of transmission modes to increase awareness for this indication. In our study, no opportunity for “before aseptic technique” was observed. As reported by the local team, this indication was most likely lacking due to the postponement of elective procedures such as vaccinations and non-emergency surgery at FRH, as well as hesitancy amongst the local population to visit healthcare facilities during the pandemic. ABHR consumption showed an overall average of 3.29 ml ABHR per consultation but varied considerably with a first large peak in February 2020, a second peak in November 2020 and a third in January to February 2021. The first peak starting could be explained by the declaration of a “Public Health Emergency of National Concern” [43] on January 30, 2020 and the first detection of a COVID-19 case at the beginning of March 2020 in Guinea. These declarations are likely to have increased awareness for HH resulting in an ABHR consumption of 23 mL per patient consultation. This initially low consumption in conjunction with high compliance rates can imply that HCWs use less ABHR than recommended for very action. A discrepancy, that was already previously observed in this setting [20]. The increase in consumption likely reflects a sensibilization of the amount needed per action. However, it may also reflect misuse, such as spilling or usage outside the healthcare facility, as reported by the local research team. The second peak could be explained by the IPC training, where every HCW received a bottle of 250 ml ABHR. The third peak can be partly explained by a further outbreak of Ebola in Guinea with the first Ebola case declared on February 14, 2021; [44], which could lead to increased awareness for HH. It should also be noted that there was a decrease in patient consultations from February to April 2020, which is likely due to potential patients’ fear of contracting SARS-CoV-2 in the hospital setting, as reported by the local research team and already described in previous studies from other settings [45, 46]. However, the decline in patient numbers can also be explained by the disrupted access to health facilities or services as a result of the COVID-19 restrictions, such as travel bans [47].

The three peaks in ABHR consumption occurred during a ‘seller’s market’, in which global ABHR demand was high while supply was low [48], meaning that resource-limited regions might not be as economically competitive and therefore have more difficulty obtaining externally soured ABHR. HCWs at the HRF, however, did not experience ABHR accessibility issues due to the available locally produced supply. This experience, therefore, highlights the importance of local ABHR production as a key pandemic preparedness strategy.

### Limitations

Our study has several limitations that need to be acknowledged. The COVID-19 knowledge and attitude questionnaires were not previously validated by global institutions such as the WHO but were locally developed and context adapted. HCWs had difficulties understanding the Likert scale of the attitude assessment at baseline. Therefore, in the first and second follow-up, the study team modified the Likert-scale by retaining the seven items and providing appropriate verbal labels to each number. This modification may have an impact on the comparability of attitude at baseline with both follow-ups. Since there was no direct communication between the triage team and the observer given that the observer was not supposed to intervene, no conclusion about the triage outcome “suspect” or “not suspect” could be drawn by the observer. We therefore cannot assess the outcome and respective measures that were taken after the triage process with this tool, but only assess the triaging process. The lack of observations of the indication “before aseptic procedure” limits its comparability with previous studies of the PASQUALE project, on the other hand emphasises the need to raise awareness during data collection.

Lastly, there was a military coup in September 2021 in Guinea during the study period, resulting in an unpredicted and sudden turnover of local study members with some loss to study data.

### Recommendations

In conclusion we can recommend: (1) Further measures need to be taken to foster pandemic preparedness and response in the FRH and HCCs. Regular IPC knowledge and practice training focusing on practical aspects should be in place and can be especially effective in HCCs. Local ABHR production ought to be established to maintain accessibility independent of global imports which are vulnerable to price spikes and shortages in a pandemic situation. Protective strategies are needed to mitigate pandemic stressors on HCWs. (2) In terms of local narratives, trainers should be aware of locally and globally circulating knowledge, especially of social media sources that might spread misinformation and be reinforced through word of mouth. (3) International assessment tools can be used in other settings, but should be adapted to the context and educational background of participants. The WHO HH observation form could be extended to include additional indications, such as “before” and “after PPE use” where applicable.

## Electronic supplementary material

Supplementary material

## Data Availability

The datasets generated and/or analysed during the current study are not publicly available due to traceability of individuals given the small sample size but are available from the corresponding author on reasonable request.

## Abbreviations

ABHR: alcohol-based handrub
ANSS: Agence Nationale de la Sécurité Sanitaire
CDC: Centers for Disease Control and Prevention
COVID-19: Coronavirus Disease 2019
CTEpi: Centre de traitement des épidemies
FRH: Faranah Regional Hospital
HCC: healthcare centres
HCW: healthcare worker
HH: hand hygiene
IPC: infection prevention and control
IQR: interquartile range
OR: Odds ratio
SARS-CoV-2: severe acute respiratory syndrome Coronavirus 2
WHO: World Health Organisation
