# Correction: Healthcare workers’ knowledge, attitude and practices on infection prevention and control in the context of the COVID-19 pandemic at the Faranah regional hospital and associated healthcare centers, Guinea

**DOI:** 10.1186/s13756-025-01566-x

**Published:** 2025-05-06

**Authors:** Lena Landsmann, Anna Borodova, Carlos Rocha, Aziz Amadou Diallo, Kamis Mamadou Diallo, Matthias Borchert, Mardjan Arvand, Mamadou Diallo, Rebekah R. Wood, Sophie A. Müller

**Affiliations:** 1 Unit for Hospital Hygiene, Infection Prevention and Control, Robert Koch Institute, Berlin, Germany; 2 Centre for International Health Protection, Robert Koch Institute, Berlin, Germany; 3 Faranah Regional Hospital, Faranah, Guinea

**Correction: Antimicrobial Resistance & Infection Control (2024) 13:79**

10.1186/s13756-024-01435-z

The authors wish to note the following two amendments to the original article:

1. In Table 4, the line and associated date for ‘HCW wearing masks’ should be disregarded as well as all related sections in the text in order to focus on the type of mask and correct usage of mask only.
2. The authors discovered an error in the Excel plotting that resulted in the graph and reporting of alcoholic-hand rub solution (ABHR) being shifted by one month. This does not impact the conclusions, but they would like to note the correction to the graph and related text accordingly to maintain accuracy.

