# Supplementary material for: Healthcare workers’ knowledge, attitude and practices on infection prevention and control in the context of the COVID-19 pandemic at the Faranah regional hospital and associated healthcare centers, Guinea
Source: Antimicrob Resist Infect Control. 2024 Jul 18;13:79. doi: 10.1186/s13756-024-01435-z (PMC11256390; doi:10.1186/s13756-024-01435-z)
Supplement: Supplementary file 1 — Supplementary material [file 13756_2024_1435_MOESM1_ESM.docx]

**Supplementary Materials**

| **Supplementary Table 1. Study population** | | |
| --- | --- | --- |
|  | | n (%) |
| **Faranah Regional Hospital** | | 32 |
| By professional categories | |  |
|  | Medical doctor | 4 (12.5%) |
|  | Auxiliary Nurse | 11 (34.4%) |
|  | Nurse | 7 (21.9%) |
|  | Midwife | 3 (9.4%) |
|  | Other* | 7 (21.9%) |
| **Health Care Centers** | | 16 |
| By professional categories | |  |
|  | Medical doctor | 1 (6.2%) |
|  | Auxiliary nurse | 11 (68.7%) |
|  | Nurse | 2 (12.5%) |
|  | Other* | 2 (12.5%) |

**Medical students, nursing students, technicians*

**Supplementary Table 1-**

| **Supplementary Table 2. Hand Hygiene Compliance before and after PPE use, Faranah Regional Hospital, Guinea** | | | | | | | | |
| --- | --- | --- | --- | --- | --- | --- | --- | --- |
| **Variable** | | Baseline | | | Second Follow-up | | | P** |
|  |  | No. of HH actions | No. (%) of HH opportunities | Compliance, % (95% CI)* | No. of HH actions | No. (%) of HH opportunities | Compliance, % (95% CI)* |  |
| **Indication** | |  |  |  |  |  |  |  |
|  | Before PPE use | 4 | 13 | 30.8 (-19.4 – 80.9) | 6 | 11 | 54.5 (-0.0 – 1.1) | 0.405 |
|  | After PPE use | 6 | 11 | 54.5 (0.0– 1.1) | 6 | 8 | 75.0 (15.0 – 1.35) | 0.519 |
| *width of CI adjusted for lack of independence by inflating standard error by a factor of 2.  ** determined by χ² test with standard error corrected by factor 2 to adjust for lack of independence | | | | | | | | |
